# Development, evaluation, and implementation of an online pain assessment training program for staff in rural long-term care facilities: a case series approach

**DOI:** 10.1186/s12877-022-03020-8

**Published:** 2022-04-18

**Authors:** Natasha Gallant, Thomas Hadjistavropoulos, Emily M. Winters, Emma K. Feere, Abigail Wickson-Griffiths

**Affiliations:** 1 Centre on Aging and Health, University of Regina, Regina, SK S4S 0A2 Canada; 2 Department of Psychology, University of Regina, Regina, SK S4S 0A2 Canada; 3 Faculty of Nursing, University of Regina, Regina, SK S4S 0A2 Canada

**Keywords:** Dementia, Implementation Science, Long-Term Care, Online Training, Pain, Rural Settings

## Abstract

**Background:**

Pain among long-term care (LTC) residents, and especially residents with dementia, is often underassessed and this underassessment has been attributed, in part, to gaps in front-line staff education. Furthermore, although evidence-based clinical guidelines for pain assessment in LTC are available, pain assessment protocols are often inconsistently implemented and, when they are implemented, it is usually within urban LTC facilities located in large metropolitan centers. Implementation science methodologies are needed so that changes in pain assessment practices can be integrated in rural facilities. Thus, our purpose was to evaluate an online pain assessment training program and implement a standardized pain assessment protocol in rural LTC environments.

**Methods:**

During the baseline and implementation periods, we obtained facility-wide pain-related quality indicators from seven rural LTC homes. Prior to implementing the protocol, front-line staff completed the online training program. Front-line staff also completed a set of self-report questionnaires and semi-structured interviews prior to and following completion of the online training program.

**Results:**

Results indicated that knowledge about pain assessment significantly increased following completion of the online training program. Implementation of the standardized protocol resulted in more frequent pain assessments on admission and on a weekly basis, although improvements in the timeliness of follow-up assessments for those identified as having moderate to severe pain were not as consistent. Directed content analysis of semi-structured interviews revealed that the online training program and standardized protocol were well-received despite a few barriers to effective implementation.

**Conclusions:**

In conclusion, we demonstrated the feasibility of the remote delivery of an online training program and implementation of a standardized protocol to address the underassessment of pain in rural LTC facilities.

**Supplementary Information:**

The online version contains supplementary material available at 10.1186/s12877-022-03020-8.

## Background

Pain is highly prevalent among residents living in long-term care (LTC) facilities [1]. Among residents with moderate to severe dementia, pain can be particularly debilitating as it can lead to behavioural disturbances, mood symptoms, and impaired physical functioning [2–4]. These pain-related behavioural disturbances are often misattributed to psychiatric causes and tend to be treated with psychotropic medications rather than analgesic medications [5]. This misattribution has adverse consequences for LTC residents with dementia since the use of psychotropic medications in this population hastens death [6]. Despite the increased prevalence of pain among LTC residents and greater adverse consequences associated with pain-related behavioural disturbances in dementia, pain continues to be underassessed and undertreated in LTC settings[7–12].

The assessment of pain among residents with dementia requires familiarity with state-of-the-art pain assessment practices [13]. That is, existing guidelines recommend that pain assessments using standardized tools are completed on admission and on a weekly basis following admission and, when residents are found to have at least moderate pain, an intervention should be documented within 24 hours with a reassessment of pain and intervention-related side effects within the next 24 hours [14]. These guidelines have been deemed desirable and feasible by LTC administrators, physicians, and nurses [15]. Furthermore, regular use of standardized tools, such as the Pain Assessment Checklist for Seniors with Limited Ability to Communicate scales (PACSLAC [16, 17]), has been shown to result in improved pain management practices, decreased pain levels as assessed by front-line staff, and reduced use of benzodiazepines [9, 18].

Despite the availability of state-of-the-art pain assessment practices, educational gaps among health professionals have been documented [19], and front-line staff working in LTC facilities frequently report that they are inadequately trained to assess pain among LTC residents with dementia [20–24]. Although pain-related knowledge gained from continuing education for LTC staff appears to improve the assessment of pain among residents in the short term [23, 25–28], this improvement in knowledge about pain needs to be accompanied by implementation science methodologies—for example, by using the Consolidated Framework for Implementation Research (CFIR [29, 30];) to guide data collection, analysis, and interpretation—so that long-term changes in pain assessment practices can be achieved [9, 31]. For example, previous research that makes use of the CFIR has demonstrated that the use of implementation science methodologies in combination with in-person pain assessment training within the LTC facility can result in an increased frequency of pain assessments using a standardized tool [9]. Seeing as this previous research was conducted in urban LTC facilities, the provision of in-person pain assessment training along with in-person visits by researchers to ensure successful implementation was very feasible. The ability to offer in-person training and visits in rural settings, however, is much less feasible.

Thus, our aim was to overcome geographic barriers to continuing education for front-line staff working in rural LTC facilities by developing and evaluating an interactive online training program focused on pain assessment practices as well as remotely implement a standardized pain assessment protocol. In applying the CFIR to our study, we implemented the core components (i.e., pain assessment training, standardized pain assessment protocol) of our intervention across all rural LTC facilities while modifying the adaptable periphery (e.g., remote delivery of pain assessment training, individualized implementation plan for standardized pain assessment protocol developed over email or telephone) of our intervention to best meet the needs of each rural LTC facility. We expected that our training and implementation approach would result in increased knowledge about pain assessment among front-line staff as well as improved pain assessment practices (reflected in both objective quality indicators and subjective front line staff reports) in each rural LTC facility.

## Methods

### Setting & Participants

LTC facilities were rural if they were located outside of census metropolitan areas and census agglomerations as defined by Statistics Canada [32]. A total of seven rural LTC facilities located in villages or towns participated in this study (Table 1). Participants for this study included directors of care, nurses, and care aides working with older adults in the participating rural LTC facilities. A total of 99 nurses and care aides across the participating rural LTC facilities completed the online training program (Table 2). Furthermore, a total of 42 directors of care, nurses, and care aides participated in the individual interviews regarding the online training program and standardized pain assessment protocol (Table 3). Nearly all full-time nurses and care aides participated in the pain assessment program since the assessment program was adopted facility-wide for quality improvement purposes.

**Table 1:** Characteristics of participating LTC facilities

Facility	A	B	C	D	E	F	G
**Municipality**	Town	Village	Town	Town	Town	Village	Town
**Number of Beds**	30	30	30	38	16	15	38

**Table 2:** Characteristics of online training program participants

Facility	A	B	C	D	E	F	G
Total	*N*=21	*N*=8	*N*=12	*N*=16	*N*=14	*N*=11	*N*=17
**Missing Data**	*N*=1	*N*=1	*N*=2	*N*=9	*N*=2	*N*=2	*N*=4
**Age**	M=44.00	M=50.00	M=40.80	M=35.86	M=40.58	M=43.78	M=42.92
	SD=11.98	SD=10.60	SD=11.68	SD=10.87	SD=14.25	SD=11.04	SD=12.60
**Experience**	M=12.65	M=18.43	M=11.40	M=6.57	M=10.92	M=10.89	M=8.15
	SD=10.06	SD=11.65	SD=9.89	SD=8.36	SD=11.10	SD=10.42	SD=7.02
**Gender**							
***Males***	*N*=1	*N*=0	*N*=0	*N*=0	*N*=0	*N*=0	*N*=1
	%=5.00	SD=0.00	SD=0.00	SD=0.00	SD=0.00	SD=0.00	SD=7.69
***Females***	*N*=19	*N*=7	*N*=10	*N*=7	*N*=12	*N*=9	*N*=12
	%=95.00	%=100.00	%=100.00	%=100.00	%=100.00	%=100.00	%=92.31
**Occupation**							
***Nurses***	*N*=7	*N*=7	*N*=7	*N*=3	*N*=8	*N*=6	*N*=4
	%=35.00	%=100.00	%=70.00	%=42.86	%=66.67	%=66.67	%=30.77
***Care Aides***	*N*=13	*N*=0	*N*=3	*N*=4	*N*=4	*N*=3	*N*=9
	%=65.00	%=0.00	%=30.00	%=57.14	%=33.33	%=33.33	%=69.23

**Table 3:** Characteristics of individual interview participants

Facility	A	B	C	D	E	F	G
Total	*N*=6	*N*=6	*N*=7	*N*=5	*N*=6	*N*=7	*N*=5
**Occupation**							
***Director of Care***	*N*=1	*N*=1	*N*=1	*N*=1	*N*=1	*N*=1	*N*=1
	%=16.67	%=16.67	%=14.29	%=20.00	%=16.67	%=14.29	%=20.00
***Nurses***	*N*=3	*N*=3	*N*=3	*N*=2	*N*=3	*N*=4	*N*=3
	%=50.00	%=50.00	%=50.00	%=40.00	%=50.00	%=57.15	%=60.00
***Care Aides***	*N*=2	*N*=2	*N*=3	*N*=2	*N*=2	*N*=2	*N*=1
	%=33.33	%=33.33	%=42.86	%=40.00	%=33.33	%=28.57	%=20.00

### Materials

#### Online Pain Assessment Training

In collaboration with an instructional designer and web developer, we created an interactive and dynamic online training platform concerning pain assessment in LTC with a focus on the use of the PACSLAC-II for persons with dementia (see Additional file 1 for more details). The online training program included six core modules that were each designed to be completed within ten to 15 minutes. An optional module, with videos, was developed to provide staff with the opportunity to practice using the PACSLAC-II. A formative knowledge quiz of 10 to 15 multiple-choice questions, based on our instructional content, was administered following Modules 1, 2, 3, and 5. Successfully completing each quiz was necessary for the participants to progress through the online program. One of the authors wrote the instructional content each of the modules and two of the authors wrote the questions for the formative knowledge quizzes.

#### Standardized Pain Assessment Protocol

In line with state-of-the-art pain assessment practices for LTC facilities [13–15, 33], the standardized pain assessment protocol invited front-line staff to (a) assess residents for pain using a standardized tool within 24 hours upon admission; (b) assess residents for pain using a standardized tool at least once per week; (c) document a treatment plan for residents with moderate-to-severe pain within 24 hours; (d) re-assess pain to evaluate the effectiveness of the treatment plan within 24 hours of its implementation; and (e) evaluate for side effects associated with the treatment plan within 24 hours of its implementation.

### Measures

#### Demographics

Before starting the online training program, participants responded to questions about age, gender, professional status (e.g., nurse, care aide), years of experience in profession, and years of experience working in LTC.

#### Knowledge Test (KT)

The KT was adapted from Gagnon et al. [34] who evaluated a video-based training program for pain assessment in LTC. The KT consisted of 14 multiple-choice questions with a single response option that was correct. For example, participants were asked whether observational pain assessment measures are most likely to identify pain a) when the patient is at rest, b) during movement, c) in the morning, or d) in the evening). Participants were provided with feedback on whether they chose the correct response option (i.e., b) during movement) or one of the incorrect response options (i.e., a) when the patient is at rest, c) in the morning, or d) in the evening). After completing all 14 multiple-choice questions from the KT, participants were provided with a percentage score based on the number of questions that they answered correctly (e.g., “You scored 85.71%”).

#### Readiness for Organizational Change (RFOC)

The RFOC, which has been used in previous research in the context of pain assessment in LTC (e.g., [35]), evaluated each LTC facility’s RFOC. The RFOC consisted of 10 items (e.g., To what extent are the interventions consistent with the values, attitudes, and beliefs of the practice environment?) rated on a 6-point Likert-type scale ranging from 0 (i.e., not at all) to 5 (i.e., a great deal). The RFOC was administered prior to starting the online training program. Cronbach’s alpha for the RFOC was .867.

#### Online Training Evaluation Questionnaire (OTEQ)

The OTEQ was adapted from Gagnon et al. [34] who evaluated a video-based training program for pain assessment in LTC. The online training questionnaire consisted of 31 items rated on a 5-point Likert scale ranging from 0 (i.e., strongly disagree) to 4 (i.e., strongly agree). The first scale evaluated participants’ perceptions of the quality of the content of the online training program (i.e., Content Quality Scale [CQS]; 16 items). This scale was further subdivided into two subscales: The first subscale evaluated perceptions of the overall value of the online training program (i.e., General–Content Quality Scale [G-CQS]; 5 items) and the second subscale evaluated perceptions of the value of the online training program for their job in their LTC facility (i.e., Specific–Content Quality Scale [S-CQS]; 7 items). Furthermore, four items assessed the degree of prior familiarity with and use of standardized pain tools. Finally, the second scale asked questions about participants’ perceptions of the quality of the technical features of the online training program (i.e., Technical Quality Scale [TQS]; 15 items). The OTEQ was administered following the completion of the online training program. Cronbach’s alphas were .741, .759, and .829 for the G-CQS, S-CQS, and TCS, respectively.

#### Quality Indicators

Facility-wide pain assessment quality indicators, which have been successfully implemented in previous research in LTC facilities [9, 12], were derived from the set of expert- and consensus-based clinical for pain assessment in LTC facilities developed in consultation with public policy and geriatric pain experts [14, 15]. Quality indicators included the (a) percentage of residents assessed for pain using a standardized tool within 24 hours upon admission; (b) percentage of residents assessed for pain using a standardized tool at least once per week; (c) percentage of residents with moderate to severe pain for which a treatment plan is implemented within 24 hours; (d) percentage of residents with moderate to severe pain for which the treatment plan is evaluated (through re-assessment) within 24 hours of being implemented; and (e) percentage of residents with moderate to severe pain for which side effects are evaluated within 24 hours of the treatment plan being implemented.

#### Semi-Structured Individual Interviews

Individual interviews were conducted over the telephone prior to and following the implementation of the online training program and standardized pain assessment protocol. Interviews were semi-structured as they followed a moderator guide designed for this study (see Additional file 2 for more details). The moderator guide included questions related to training in pain assessment and management, experiences with pain assessment and management in LTC, the online training program, implementation of the standardized pain assessment protocol, and issues of relevance to pain assessment and training in rural LTC settings.

#### Procedure

The study took place over three phases (Table 4). Following approval of our institutional ethics review board, directors of care working within rural LTC facilities were informed about the training program and implementation protocol and were asked if they would be interested in implementing the training and pain assessment protocol as a means of raising standards of care for the LTC facility. These LTC facilities were approached by regional LTC administrators. Facilities implemented the online pain assessment and management training program and recommended standardized pain assessment protocol as part of their quality improvement processes. Staff members who were given access to the online training were also provided with the opportunity to also participate in the study by consenting to the completion of research questionnaires. Furthermore, directors of care provided suggestions of qualifying staff members who could participate in individual interviews regarding the online training program and standardized pain assessment protocol. These staff members provided separate consent for participating in individual interviews that were audio recorded for the purpose of transcription. For each of these opportunities to participate in the study, staff members were informed that participation was voluntary and would in no way affect their position within the facility or their employment status.

**Table 4:** Description of the study’s methodology

• Baseline (8 weeks)	
° No changes to current pain assessment protocol.	
° Directors of care collected weekly quality indicator data via chart reviews.	
° Interviews completed with director of care, nurses, and care aides.	
• Training (8 to 24 weeks)	
° Pre-training questionnaires completed.	
° Web-based training program completed.	
° Post-training questionnaires completed.	
° Implementation plan developed.	
• Implementation (8 weeks)	
° Implementation of the standardized pain assessment protocol.	
° Directors of care collected weekly quality indicator data via chart reviews.	
° Interviews completed with director of care, nurses, and care aides.	

## Results

### Online Training Program

On average, the core modules of the program took just over two hours to complete (M = 130.71, SD = 51.12, Median = 124.26, IQR = 90.86 – 156.49). With regards to the knowledge quizzes completed after Modules 1, 2, 3, and 5, all LTC staff answered at least 75% of the multiple-choice questions correctly. On average, staff answered almost 95% of questions correctly confirming their attendance to the material (M = 94.48, SD = 6.04, Median = 95.00, IQR = 95.00 – 100.00). A breakdown of the length of time to complete each module and KT scores is provided in Additional file 1.

### Knowledge Test (KT)

A Shapiro-Wilk test of normality on the difference scores for the KT indicated that our data were not normally distributed, *p* = .000. Thus, non-parametric statistical analyses were performed. A Wilcoxon signed-rank test showed that staff achieved significantly higher percentage scores immediately following the online training program (Md = 93.00, IQR = 86.00–100.00, *N* = 65) compared to prior to starting the online training program (Md = 71.00, IQR = 64.00–86.00, *N* = 70), *Z* = -5.228; *p* = .000.

### Readiness for Organizational Change (RFOC)

Across facilities, the average RFOC score was 3.51 (SD = 0.58). Means and standard deviations for average RFOC scores by facility are presented in Table 5. Facility D, E, and F had average RFOC scores that were above the average RFOC score across facilities, whereas Facility A, B, C, and G had average RFOC scores below the average RFOC score across facilities.

**Table 5:** Means and standard deviations for average RFOC scores by facility

Facility	A	B	C	D	E	F	G
**RFOC**^**a**^	*N*=20	*N*=7	*N*=8	*N*=4	*N*=12	*N*=9	*N*=13
	M=3.44	M=3.50	M=3.28	M=3.60	M=3.73	M=3.60	M=3.45
	SD=0.68	SD=0.43	SD=0.54	SD=0.42	SD=0.38	SD=0.51	SD=0.74

^a^Readiness for Organizational Change

### Online Training Evaluation Questionnaire (OTEQ)

Average G-CQS, S-CQS, and TQS scores were 3.21 (SD = 0.46), 3.22 (SD = 0.55), and 2.82 (0.40), respectively. Means and standard deviations for average G-CQS, S-CQS, and TQS scores by facility are presented in Table 6. Facility E had average G-CQS, S-SQS, and TQS scores that were below the average G-CQS, S-CQS, and TQS scores across facilities. Facility D, F, and G had some average scores above and other average scores below the average G-CQS, S-CQS, and TQS scores across facilities. Facility A, B, and C had average G-CQS, S-CQS, and TQS scores that were above the average G-CQS, S-CQS, and TQS scores across facilities.

**Table 6:** Means and standard deviations for average G-CQS, S-CQS, and TQS scores by facility

Facility	A	B	C	D	E	F	G
**G-CQS**^**a**^	*N*=19	*N*=6	*N*=6	*N*=6	*N*=11	*N*=5	*N*=10
	M=3.39	M=3.47	M=3.47	M=3.17	M=2.67	M=3.12	M=3.28
	SD=0.52	SD=0.45	SD=0.39	SD=0.48	SD=0.43	SD=0.82	SD=0.40
**S-CQS**^**b**^	*N*=19	*N*=6	*N*=6	*N*=6	*N*=11	*N*=5	*N*=10
	M=3.33	M=3.29	M=3.26	M=3.31	M=2.81	M=3.37	M=3.21
	SD=0.45	SD=0.40	SD=0.42	SD=0.49	SD=0.43	SD=0.46	SD=0.45
**TQS**^**c**^	*N*=19	*N*=6	*N*=6	*N*=6	*N*=11	*N*=5	*N*=10
	M=2.87	M=3.09	M=3.00	M=2.81	M=2.47	M=2.87	M=2.80
	SD=0.37	SD=0.47	SD=0.38	SD=0.35	SD=0.26	SD=0.34	SD=0.45

^a^General–Content Quality Scale.

^b^Specific–Content Quality Scale.

^c^Technical Quality Scale.

### Quality Indicators

As shown in Table 7, implementation of the standardized protocol resulted in more frequent pain assessments on admission and on a weekly basis, although improvements in the timeliness of follow-up tasks for those identified as having moderate to severe pain were not as consistent. Following implementation of the standardized protocol, the frequency of weekly pain assessments for current residents continued to improve for Facilities A, B, and E and improvements remained relatively constant for Facilities D and G over the 8-week implementation period. While the frequency of weekly pain assessments for current residents decreased over the 8-week implementation period for Facility C (i.e., 100% to 84%) and Facility F (i.e., 53% to 33%), these pain assessments were still much more frequent compared to the baseline period (i.e., 0%). With regards to follow-up tasks for residents identified as having moderate to severe pain, improvements following the implementation of the standardized pain assessment protocol tended to either continue or remain relatively stable over the course of the implementation period. The only exception to this trend was Facility C as the percentage of assessments for side effects for residents with moderate to severe pain went from 60% during the first week to 0% during the remaining weeks of the implementation period.

**Table 7:** Quality indicators averaged over the 8-week baseline and implementation periods

Facility	A	B	C	D	E	F	G
**QI 1**^**a**^							
***Baseline***	%=100.00	%=50.00	%=100.00	%=66.67	%=—	%=0.00	%=100.00
***Implementation***	%=100.00	%=0.00	%=100.00	%=100.00	%=—	%=—	%=100.00
**QI 2**^**b**^							
***Baseline***	%=6.70	%=73.36	%=0.00	%=0.00	%=4.69	%=0.00	%=10.63
***Implementation***	%=64.78	%=80.53	%=88.37	%=98.03	%=51.11	%=35.00	%=99.34
**QI 3**^**c**^							
***Baseline***	%=73.68	%=91.30	%=87.50	%=87.50	%=10.00	%=58.33	%=93.62
***Implementation***	%=35.48	%=100.00	%=42.86	%=70.00	%=66.67	%=87.50	%=85.71
**QI 4**^**d**^							
***Baseline***	%=31.58	%=84.78	%=0.00	%=0.00	%=16.67	%=0.00	%=53.19
***Implementation***	%=6.45	%=100.00	%=50.00	%=60.00	%=66.67	%=75.00	%=71.43
**QI 5**^**e**^							
***Baseline***	%=0.00	%=77.17	%=12.50	%=12.50	%=13.33	%=16.67	%=41.49
***Implementation***	%=0.00	%=97.14	%=21.43	%=30.00	%=0.00	%=0.00	%=28.57

^a^New residents assessed using the PACSLAC-II on admission averaged across the total number of weeks from each period.

^b^Current residents assessed using the PACSLAC-II at least once per week averaged across the total number of weeks from each period.

^c^Residents with moderate-to-severe pain with a treatment plan within 24 hours averaged across the total number of weeks from each period.

^d^Residents with moderate-to-severe pain with a reassessment within 24 hours averaged across the total number of weeks from each period.

^e^Residents with moderate-to-severe pain assessed for side effects within 24 hours averaged across the total number of weeks from each period.

### Semi-Structured Individual Interviews

For the interview portion of the study, the qualitative descriptive approach that we employed is an approach commonly used to examine healthcare-related phenomena [36, 37]. That is, narrative responses from individual interviews were subjected to directed content analysis using a template organizing style of interpretation [38, 39]. Furthermore, findings from the directed content analysis were supplemented with descriptive quantitative data to support a mixed methods approach (e.g., [40]).

As directed content analysis is an iterative process, the qualitative analyses were conducted in two phases. For the first phase, a first coder and a second coder categorized narrative responses with the use of a code manual based on the interview moderator guide. Once the first and second coders familiarized themselves with the content of the interviews, several steps were taken to produce a finalized code manual [41–46]. The finalized manual contained ten thematic categories organized into five families of codes.

To ensure that our directed content analysis was reproducible, we conducted a series of analyses to ensure intercoder reliability and consensus agreement [47]. That is, using the finalized code manual, a total of 1,510 narrative responses were identified and organized into these thematic categories by the first coder. Subsequently, a third coder organized all pre-determined blocks of narrative responses into these thematic categories. A calculation of Cohen’s kappa comparing the organization of narrative responses into thematic categories by the first and third coders was used to assess intercoder reliability [48]. Overall, satisfactory agreement between the original and reliability coders was observed when a Cohen’s kappa was calculated, κ = .794, *p* = .000. Using negotiated agreement, consensus was reached between the first and third coders for all 258 of the identified disagreements. The original coder deferred to the reliability coder 41.86% of the time and the reliability coder deferred to the original coder 58.14% of the time.

The analysis of interviews revealed five themes: (1) confidence resulting from previous training experiences; (2) communication and interpretation patterns in current practices; (3) technology as a way of learning and impressions of learning through technology; (4) competing demands in implementing changes; and (5) resources in rural settings. The frequency of and representative quotes for each of the themes and subthemes are detailed in Additional file 3. With regards to confidence resulting from previous training experiences, two subthemes emerged. The first subtheme described *a lack of confidence due to limited training experiences*. When asked how they felt about the level of training in pain assessment and management that they have received, most nurses and care aides reported that they had received no or little formal education on pain assessment and management. Compared to nurses, care aides were more likely to report that they had received no formal education in pain assessment and management. Nurses noted that their formal education was usually limited to being provided with observational pain tools with minimal guidance on how to implement these tools in LTC settings. The second subtheme described *a sense of confidence due to adequate training experiences*. Some nurses and care aides reported that, for the most part, they felt comfortable and confident in their ability to assess and manage pain in LTC settings. Some, but not most, participants explained that they had received basic education in pain assessment and management as part of their formal education followed by informal training while they were on the job. Examples of on-the-job training included learning to read residents’ facial expressions and body language, being provided with and asked to use observational pain assessment tools (e.g., PACSLAC-II [16, 17]; Pain Assessment in Advanced Dementia [PAINAD [49]), and attending continuing education workshops and conferences related to palliative care or pain management. Greater comfort and confidence in pain assessment and management seemed to be associated with more years of experience in LTC settings.

With regards to communication and interpretation patterns in current practices, two subthemes emerged. The first theme described *ineffective communication and inaccurate interpretation of expressed needs*. Subjectivity and variability in pain assessment and management strategies were identified as major limitations of pain practices for LTC residents. A lack of communication was noted to be a barrier to effective pain assessment and management practices. Several nurses and care aides explained that it was more difficult to assess and treat for pain among residents with dementia or among residents with chronic pain. Another commonly reported challenge was to find a balance between using pharmacological interventions to manage pain while also ensuring that residents’ quality of life is not compromised by the side effects of these pharmacological interventions. Finally, a few staff members noted that they felt that pain is sometimes minimized by other staff members because of the belief that pain is a natural part of aging and that it therefore cannot be managed. The second theme described *effective communication and accurate interpretation of needs*. For example, the importance of pain assessment and management in improving the quality of life of LTC residents was highlighted by several care aides, nurses, and directors of care. Many nurses and care aides pointed to the key role that communication played in successfully assessing and managing residents’ pain. Moreover, some nurses indicated that their practices were better for residents who were in acute pain or who were experiencing pain within the context of palliative care.

With regards to technology as a way of learning and impressions of learning through technology, four subthemes emerged. The first subtheme involved *rejecting technology as a way of learning*. A few staff members indicated a preference for in-person learning within a classroom environment given their beliefs that it would result in better engagement through discussions with peers and by being able to ask in-the-moment questions to the facilitator. One of the major challenges to learning in an online environment was a lack of time to complete training during their shifts. Other challenges included having an unstable Internet connection, being unable to access a computer equipped with speakers at work, needing to attend to higher priority items while at work, and having a sense of discomfort with technology. The second subtheme involved *embracing technology as a way of learning*. One of the most cited facilitators was that staff were becoming more comfortable with online training as they have had more exposure to online training in recent years and understand that online training is likely to become more commonly used. The convenience of being able to access online training at a time that best suited the staff member was also highlighted as an advantage. The third subtheme described *positive impressions of learning through technology*. Most staff members felt that the online training program left them with positive impressions of learning through technology was easy to understand and useful to their jobs. The fourth subtheme described *negative impressions of learning through technology*. While a few care aides felt that the material was too complex to be useful, some nurses noted that the material was too simple to be useful. Some staff members also felt that the online training program was too lengthy.

With regards to competing demands in implementing changes, two subthemes emerged. The first theme described *the ease of implementing changes due to alignment with current practices.* One of the identified facilitators included a willingness or open-mindedness towards changes in practices such as pain assessment and management practices. Support from management regarding practice changes was another identified facilitator. Once the standardized pain assessment and management protocol was implemented, staff reported that it was beneficial to be able to see improvements among residents. That is, residents were reportedly happier and more comfortable because their pain was better assessed and managed. Staff also reported fewer aggressive behaviours by residents once their pain was better managed. For the most part, staff members felt that the standardized pain protocol was straightforward to implement. Integrating pain assessments into an already established weekly routine (e.g., baths) and charting process (e.g., daily records) was perceived as a facilitator. The second theme described *the challenges faced when trying to implement change*. One of the most reported perceived barriers was miscommunications or a lack of communication across professions or from one shift to the next. The subjectivity of standardized pain assessment tools was identified as another challenge as most staff had hoped to be provided with a more objective way of assessing pain. Another challenge involved a lack of time to regularly assess each resident for pain on a weekly basis and to complete a follow-up pain assessment for residents with moderate-to-severe pain. Finally, a few staff members reported that some of their colleagues were generally resistant to changes in practices and that changes in pain assessment practices were also slow for those colleagues.

With regards to resources in rural settings, two subthemes emerged. The first theme described *limited access to resources*, including limited access to training opportunities in pain assessment and management as well as services to support current pain assessment and management practices. The second theme described *ways of overcoming limited access to resources*. A few care aides, nurses, and directors of care spoke of the benefits of working in a rural LTC facility. More specifically, staff highlighted the fact that they can provide better quality of care for residents because they can check with residents more frequently and spend more one-on-one time with the residents when completing assessments. Some of the factors that facilitated the benefits of working in rural LTC facilities included adequate internet connectivity, regular physician visits, and access to a local pharmacy. Online training was reported to be beneficial for LTC staff working in rural settings as it increased opportunities for continuing education. Rural LTC staff also identified in-service training by out-of-town clinical nurse educators or accessibility of training opportunities in nearby mid-sized metropolitan cities as benefits.

## Discussion

Our study provides support for the use of an online approach to continuing education and remote delivery of implementation strategies in rural LTC facilities. Our results confirmed that, once front-line staff completed our online training program, the remote implementation of a related standardized protocol resulted in notable improvements in the frequency of pain assessments. Specifically, we saw dramatic increases in the number of patients assessed for pain on a weekly basis. For example, prior to our online training program and standardized protocol, Facility D did not assess any of their residents for pain using a standardized tool on a weekly basis (i.e., 0.00%). By the end of our study, Facility D was assessing almost all their current residents for pain (i.e., 98.03%). Similar increases were seen across all other facilities (e.g., 6.70% to 64.78% for Facility A). It is noteworthy that the frequency of these assessments increased without additional staffing resources and that most front-line staff indicated that the standardized protocol was feasible. The areas where we could have seen greater consistency in benefit across facilities were treatment plan documentation, reassessment following treatment plan, and assessment of side effects following treatment. Future studies should place greater emphasis on these indicators in implementation plans.

Our results also confirmed that our online training program, specifically designed for front-line staff working in rural LTC facilities, was successful in addressing educational gaps regarding state-of-the-art pain assessment practices. That is, we were able to document significant increases in pain assessment knowledge across LTC facilities. Beyond increases in knowledge, front-line staff also reported that they were more confident assessing pain in individuals with cognitive impairments such as dementia. The online training program was described as useful, helpful, practical, and valuable. Although some front-line staff indicated that the online training program was too long, the information presented was deemed clear, organized, interesting, and sufficient.

Prior to completing the online training program, most nurses and care aides in the study reported having received little to no formal training in pain assessment and management for LTC residents. Previous studies have also identified the limited availability of formal training in pain assessment and management training among healthcare professionals [19–24]. Although several nurses and care aides in this study reported a strong interest in formal training opportunities regarding pain assessment and management, barriers to completing the online training program were nevertheless identified. The most noteworthy barrier identified was a lack of time. This finding is in line with previous research showing that care tasks are becoming more frequently missed or rushed among LTC staff who are also experiencing increased rates of burnout and job dissatisfaction [50–52]. It is likely that this reported lack of time is evidence of the need for the development and implementation of jurisdictional policies that prioritize the assessment and management of pain within the LTC sector. That is, the absence of evidence-based legislative and regulatory standards related to pain assessment and management in LTC facilities likely contributes to the reported lack of time to complete related training programs [53].

Moreover, front-line staff working in LTC facilities reported communication as a critical component in implementing evidence-based pain assessment and management practices. That is, front-line staff who spoke positively about pain practices in their LTC facility often attributed this success to effective communication between staff members, whereas front-line staff who spoke negatively about pain practices in their LTC facility often noted that poor communication was a primary barrier to good pain practices. Communication was also deemed important for the successful implementation of the standardized pain assessment protocol. Specifically, a lack of communication from one shift (e.g., day shift) to the next shift (e.g., night shift) or from one group of staff (e.g., nurses) to another group of staff (e.g., care aides) was one of the most reported barriers for implementing the protocol. Success of this can be facilitated with interdisciplinary collaboration including collaboration with researchers [54, 55].

### Limitations and Directions for Future Research

The present study included a baseline and implementation period but did not involve an extended follow up period. It would be important for future research to examine whether implemented changes are sustained beyond the active implementation process. Although the inclusion of staff perspectives is crucial to implement changes such as those related to pain assessment and management, future studies should consider incorporating further perspectives. For example, a better understanding of changes that residents and their family members observe might provide useful insights into the implementation process in LTC settings. Moreover, selection biases may have affected the generalizability of results as our study did not involve a random selection of rural facilities.

While the present study used the CFIR as part of an implementation study, it might be beneficial for future research to employ the CFIR prior to implementation as it allows for barriers to be addressed and facilitators to be leveraged before the introduction of an intervention [30, 56–59]. A pre-implementation approach may also provide opportunities for co-creation of the intervention with relevant stakeholders such as LTC residents, front-line staff, and administrators [60, 61]. For example, given that communication was underscored as a key contributor to improving pain practices, the online training program in the present study could be modified to specifically address ways in which front-line staff can more effectively communicate across shifts and groups of staff. Using a pre-implementation and co-creation approach would allow for these types of observations to be considered at the same time as the intervention is being developed so that such modifications could be made prior to the implementation.

## Conclusions

Using implementation science methodologies, our study provides evidence to support the feasibility of remotely implementing an online training program and a standardized protocol to better assess for pain among LTC residents living in rural settings. Lessons learned from this study could be applied to the implementation of other remote interventions that could improve the frequency and timeliness of pain assessments in LTC facilities in rural settings across Canadian jurisdictions. Increasing the frequency and timeliness of pain assessments in LTC facilities is expected to result in improved pain management which, in turn, would lead to reductions of pain-related behavioural disturbances among LTC residents with dementia. It is hoped that these advancements in pain assessment and management will lend themselves to improved quality of life for LTC residents as well as improved quality of care provided by staff working in the residents’ LTC home.

## Supplementary Information

**Additional file 1.** Overview of the Online Training Program. A description of each module of the online training program, including its title, indication of the inclusion or not of a knowledge test, time in minutes to complete, and percent correct for knowledge tests.

**Additional file 2.** Moderator Guide for Semi-Structured Interviews. A list of general questions as well as questions specific to the online training program and standardized protocol asked as part of semi-structured interviews during the baseline and implementation periods.

**Additional file 3.** Frequency of and Representative Quotes for Themes and Subthemes. A presentation of the frequency with which themes and subthemes were identified in the interview data as well as quotes that were most representative of those themes and subthemes.

## Data Availability

The data collected and analyzed as part of the present study are not publicly available due to participant confidentiality but may be available from the corresponding author upon reasonable request.

## Abbreviations

CFIR: Consolidated Framework for Implementation Research
CSQ: Content Quality Scale
G-CSQ: General–Content Quality Scale
KT: Knowledge Test
LTC: Long-term care
OTEQ: Online Training Evaluation Questionnaire
PACSLAC: Pain Assessment Checklist for Seniors with Limited Ability to Communicate
RFOC: Readiness for Organizational Change
S-CQS: Specific–Content Quality Scale
TQS: Technical Quality Scale
