# Supplementary material for: Development, evaluation, and implementation of an online pain assessment training program for staff in rural long-term care facilities: a case series approach
Source: BMC Geriatr. 2022 Apr 18;22:336. doi: 10.1186/s12877-022-03020-8 (PMC9016985; doi:10.1186/s12877-022-03020-8)
Supplement: Supplementary file 1 — Additional file 1. Overview of the Online Training Program. A description of each module of the online training program, including its title, indication of the inclusion or not of a knowledge test, time in minutes to complete, and percent correct for knowledge tests. [file 12877_2022_3020_MOESM1_ESM.pdf]

Additional File 1

Overview of the Online Training Program

Additional file 1.1 Description of each module from the online training program.

	Description	Knowledge Test
**Module 1**	Introduction	Yes
**Module 2**	Scales and Measures	Yes
**Module 3**	Limited Ability to Report Pain	Yes
**Module 4**	Coding Guidelines for the PACSLAC-II	No
**Module 5**	Practical Approaches to Assessing Pain	Yes
**Module 6**	Pain Assessment Recommendations	No
**Module 7**	PACSLAC-II Practice (Optional)	No

Additional file 1.2 Time in minutes to complete each module and percent correct for knowledge quizzes at the end of select modules.

	Time in minutes to complete	Percent correct for knowledge quizzes
**Module 1**	M=13.93	M=93.48
	SD=9.83	SD=9.54
	Md=10.06	Md=100.00
	IQR=7.97–14.19	IQR=90.00–100.00
**Module 2**	M=26.40	M=88.53
	SD=19.76	SD=8.67
	Md=19.03	Md=88.00
	IQR=14.86–26.40	IQR=81.00–94.00
**Module 3**	M=18.79	M=94.82
	SD=10.49	SD=6.69
	Md=15.86	Md=100.00
	IQR=11.98–20.01	IQR=92.00–100.00
**Module 4**	M=11.67	—
	SD=9.04	—
	Md=8.66	—
	IQR=6.15–12.83	—
**Module 5**	M=29.66	M=94.84
	SD=17.57	SD=6.91
	Md=21.89	Md=100.00
	IQR=19.25–33.16	IQR=93.00–100.00
**Module 6**	M=8.85	—
	SD=3.64	—
	Md=8.36	—
	IQR=6.62–9.43	—
**Module 7**	M=21.41	—
	SD=11.39	—
	Md=20.73	—
	IQR=13.46–25.63	—
