# Supplementary material for: Development, evaluation, and implementation of an online pain assessment training program for staff in rural long-term care facilities: a case series approach
Source: BMC Geriatr. 2022 Apr 18;22:336. doi: 10.1186/s12877-022-03020-8 (PMC9016985; doi:10.1186/s12877-022-03020-8)
Supplement: Supplementary file 2 — Additional file 2. Moderator Guide for Semi-Structured Interviews. A list of general questions as well as questions specific to the online training program and standardized protocol asked as part of semi-structured interviews during the baseline and implementation periods. [file 12877_2022_3020_MOESM2_ESM.pdf]

Additional File 2

Moderator Guide for Semi-Structured Interviews

Additional file 2.1 Moderator guide for semi-structured interviews conducted during the baseline period

General Questions

1. What has been your experience with regard to training in pain assessment in long-term care among individuals with dementia?
2. Describe how you felt with regard to the level of training you received in pain assessment prior to completing this online training?
3. Is pain assessment in long-term care an important topic to be covered in training programs or continuing education? Please explain.
4. Describe in what ways you believe pain to be adequately or inadequately addressed in your facility.
5. What role do you play in assessing pain among residents in your facility?

Online Training Questions

6. How do you feel about training that will be delivered in an online format?

Implementation Questions

7. What are particular barriers or challenges to changing or improving pain assessment practices within your facility?
    a. Personal barriers?
    b. Barriers within the facility?
    c. Structural barriers?
8. What are supporting factors that assist in changing or improving pain assessment practices within your facility?
    a. Personal supportive factors?
    b. Supportive factors within the facility?
    c. Structural supports?
9. How are decisions about changes in pain assessment practices made within your facility?
10. What role do you play in the decisions made about pain assessment practices?

Additional file 2.2 Moderator guide for semi-structured interviews conducted during the implementation period

Online Training Questions

1. What are your opinions or thoughts about the online training?
2. Describe in what ways you did or did not find this online training helpful.
3. How do you believe this online training could have been improved?
4. In terms of your current position, how would you describe the usefulness of the information shared in this online training?
5. At what stage of training would this online training be most beneficial
6. What group of individuals or staff members do you think would benefit most from completing this online training?
7. Given that you have received online training, what are your thoughts on delivering online training in a rural setting?

Implementation Questions

8. Why would you or why would you not consider implementing the practices described in the online training?
9. Describe in what ways would or would not it be feasible to implement the practices described in the online training in your facility?
