# Supplementary material for: Development, evaluation, and implementation of an online pain assessment training program for staff in rural long-term care facilities: a case series approach
Source: BMC Geriatr. 2022 Apr 18;22:336. doi: 10.1186/s12877-022-03020-8 (PMC9016985; doi:10.1186/s12877-022-03020-8)
Supplement: Supplementary file 3 — Additional file 3. Frequency of and Representative Quotes for Themes and Subthemes. A presentation of the frequency with which themes and subthemes were identified in the interview data as well as quotes that were most representative of those themes and subthemes. [file 12877_2022_3020_MOESM3_ESM.pdf]

**Additional File 3**

**Frequency of and Representative Quotes for Themes and Subthemes**

**Additional file 3.1** Representative quotes for the theme of confidence in previous training experiences

- A lack of confidence due to limited training experiences (N=159)
    - "We haven't received a whole lot of pain assessment training." (Care Aide)
    - "What I've seen is that there isn't a lot of training. What we'll have is we'll get some forms, and they might just summarize over it and then it's up to the nurse in charge to kind of train or show the other staff members, so there is no big training for it." (Nurse)
    - "It probably is inadequate unless you are going to take it upon yourself to sign up for extra education." (Director of Care)
- A sense of confidence due to adequate training experiences (N=69)
    - "I'm fairly confident. I mean, it changes every day. Our medications change daily and the way we give them changes daily. I mean, yeah, it's an ongoing process. But I mean, you're always fairly confident with what you're doing." (Nurse)
    - "Learned some of it through school and a lot of it through experience." (Care Aide)
    - "I have gone to the palliative care workshops where they've talked about pain management." (Nurse)

**Additional file 3.2** Representative quotes for the theme of communication and interpretation patterns in current practices

- Ineffective communication and inaccurate interpretation of expressed needs (N=216)
  - "...It would help if [the nurses] didn't really question us. Because by the time we're done with [the residents] and then [the nurses] have time to go see them, [the residents] are not in pain really anymore. If, when we tell [the nurses] something, they would actually go and see right away, it would be much easier. Then they would actually see that they were in pain." (Care Aide)
  - "And sometimes, you can say something to the nurse, and they're busy doing something. They're just like, "Okay, we'll get to them," and, like, they shove it off a little bit. Like, I think pain should be addressed right away." (Care Aide)
  - "I think there is a bit of problem because of consistency because—and it's going to be that way all the time because you've got different staff coming on—you've got a different nurse. I'm here for two 12-hour days and then two 12-hour nights kind of thing and then someone else comes on and I can communicate to them but it's still, you know, they're assessing things from their judgement, from their eyes, right?" (Nurse)
- Effective communication and accurate interpretation of expressed needs (N=201)
  - "Well, if I notice that there's pain or something, I go right to the nurse and tell them, and they usually act on it... usually do act on it very, very quickly." (Care Aide)
  - "Well, if we don't recognize it, the care aides would come to us and certainly bring it to our attention if [residents] are complaining of pain or if they're feeling pain and then we go in as the nurse and assess the situation to see, you know, what's hurting and then check their PRNs to see what we can give them to make them more comfortable." (Nurse)
  - "I think when those acute situations come up—somebody has a headache, somebody has a toothache—I think we are pretty good at zoning in on that. Usually, it is somebody that is cognitively aware and can communicate that to us. We are good in that we give them Tylenol and the headache goes away and the toothache goes away." (Director of Care)

**Additional file 3.3** Representative quotes for the theme of technology as a way of learning and impressions of learning through technology

- Rejecting technology as a way of learning (N=98)
  - "I am a better learner if I have someone who is talking about it. I am not a very good learner of reading and I find that, in a classroom atmosphere, I learn more because there can be discussions. If there's a misunderstanding about something, you have an opportunity to ask a question." (Care Aide)
  - "Maybe check ahead of time for a place where we can do that because, I think, I'm not sure which computer it is, but there's a couple of computers where we don't have speakers. We have to have speakers for that, right?" (Nurse)
- Embracing technology as a way of learning (N=45)
  - "I think it would be okay because we are of a society now where everything is online." (Nurse)
  - "[Pain assessment] is somewhat subjective, but as we practice, we will improve, but maybe it is better than just saying someone is having pain or whatever. At least we have a better basis for assessing it." (Nurse)
- Positive impressions of learning through technology (N=143)
  - "I think the online training was quite good." (Care Aide)
  - "I like the way that it had been set up. There were some extra things a person could click on and read on their own. I think that it was very well done." (Care Aide)
  - "I think it was good; I think it was useful and a very good refresher you know on pain and assessing it and so on." (Nurse)
- Negative impressions of learning through technology (N=59)
  - "It did take me a few sittings. I think it would have been nice to have done it right through from beginning to end but that I think is all dependent on our employer. We just kind of did it as we had time that permitted." (Care Aide)
  - "I didn't find it overly informative for myself." (Director of Care)
  - "I guess it was somewhat helpful but some of it was a little bit repetitive." (Nurse)

**Additional file 3.4** Representative quotes for the theme of competing demands in implementing changes

- The ease of implementing changes due to alignment with current practices (N=226)
    - "It's just, like, once you start doing it. It's just like second nature." (Care Aide)
    - "So, I guess with the implementation of the once a week, it is a good habit to get into so that we don't end up with someone slipping through the cracks." (Care Aide)
- The challenges faced when trying to implement change (N=171)
    - "Just communication, I think. We need to work on the communication part." (Nurse)
    - "Yeah, time is always an issue. Some days, of course, are better and some days are just worse, but I think we deal with it pretty good." (Nurse)

**Additional file 3.5** Representative quotes for the theme of resources in rural settings

- Limited access to resources (N=77)
  - "And sometimes that pain management from hospital to here its not always perfect. Like, sometimes I guess the physicians don't realize they're coming into long-term care where we don't have access to a lot of things, especially in the rural communities, because we might have a drugstore Monday to Friday, but that drugstore will not always carry the medication we need, you know." (Nurse)
  - "We're in rural. We have less access to what urban settings have, so a lot is on our shoulders." (Nurse)
  - "I guess a rural setting affects training because we don't have educators right in our facility, so we usually have to travel for education." (Nurse)
- Ways of overcoming limited access to resources (N=24)
  - "As far as like receiving or getting pain management, I think we do okay. Like we have a pharmacy here, so we are able to access them for pain management medications." (Director of Care)
  - "I think the one nice thing is we only have 15 residents at our facility and so, I mean, it can be a busy day some days, but you do actually have a lot of time to really spend with them to really, you know, talk to them and spend that one-on-one time with them." (Nurse)
